# MiR‐101 inhibits ovarian carcinogenesis by repressing the expression of brain‐derived neurotrophic factor

**DOI:** 10.1002/2211-5463.12257

**Published:** 2017-08-29

**Authors:** Ying Xu, Lei Xu, Jianbin Zheng, Lei Geng, Shuping Zhao

**Affiliations:** ^1^ Department of Gynecology Qingdao Women's and Children's Hospital Qingdao University China; ^2^ Department of Gynaecology Zibo Maternity and Child Health Hospital China

**Keywords:** exosome, invasion, migration, miR‐101, ovarian cancer

## Abstract

Ovarian cancer is one of the most lethal malignant gynecological tumors as a result of difficulties in early‐stage detection and a lack of effective treatments for patients with advanced or recurrent cancer. In the present study, we aimed to explore whether some of the microRNA (miRNA) content of serum might be related to ovarian cancer, as well as the role of these miRNAs and their intercellular transport via exosomes in ovarian cancer. We first detected the expression of six candidate miRNAs in ovarian cancer tissues and adjacent nontumor ovarian samples from 36 patients and confirmed the altered expression of four miRNAs. The level of these six candidate miRNAs was also examined in exosomes from patient serum samples. Only the level of miR‐101 was altered in both ovarian tissue samples and serum exosomes. After prediction using online bioinformatics tools and confirmation by dual‐luciferase assay and immunoblotting, we identified that miR‐101 can repress the expression of brain‐derived neurotrophic factor by targeting its 3′‐UTR. Using Transwell assays, we examined the effect of miR‐101 on migration and invasion capacity of ovarian cancer cells. The results indicated that the reduction of miR‐101 is mostly related to significant enhanced ovarian cancer cell migration. Thus, the results of the present study indicate that miR‐101 content in serum exosomes has potential as a marker for diagnosis of ovarian cancer and that miR‐101 mimics are potential therapeutic drugs for the treatment of ovarian cancer.

**Abbreviations**
BDNF: brain‐derived neurotrophic factor
MiR: microRNA

Ovarian cancer is one of the most lethal malignant gynecological tumors and its lethality mainly depends on difficulties with respect to early‐stage detection and a lack of effective treatments for patients with an advanced or recurrent status. Therefore, there is a strong need to identify new prognostic and predictive markers that enable its early diagnosis and also help to optimize and personalize treatment.

MicroRNAs (miRNA) are a group of short noncoding RNAs that are highly conserved among a wide range of species and they are generally involved in post‐transcriptional gene regulation. miRNAs play crucial roles in maintaining the homeostasis of normal human body and an altered miRNA level has been confirmed to be related to human diseases ranging from psychiatric disorders to malignant cancers 1, 2, 3. There are reports that the dysfunction of miRNAs in gynecological disorders is increasingly being recognized, such as for abortion, polycystic ovary syndrome and premature ovarian failure 4, 5, 6, 7. Although RNases Drosha and Dicer were not significantly deregulated in ovarian cancer patients, disturbed miRNA expression has been found to be related to the carcinogenesis of ovarian cancer 8, 9.

Exosomes are extracellular vesicles with a size ranging from 40 to 100 nm in diameter. They are secreted by cells in a proposed mechanism by which secreted cells pass signals to targeted cells. Tumor‐derived exosomes are functional and there is evidence to suggest that they are essential in tumor migration and that metastases such as angiogenesis are enhanced by exosomes. Altered exosomal miRNAs in serum are also found in patients with several types of cancer 10, 11.

In the present study, the expression of six candidate miRNAs was quantified in the tissue and serum exosome samples of 36 ovarian cancer patients. The function of miR‐101 during the pathogenesis of ovarian cancer was examined.

## Materials and methods

### Sample collection

All experiments were conducted with the approval of the review board for human research of Zibo Maternity and Child Health Hospital. Serum and tissue samples were collected between January 2013 and July 2016 from 36 ovarian cancer patients who underwent surgery and had not been subjected to chemotherapy before surgery. The characteristics of patients with OA (ovarian adenocarcinoma) are shown in Table 1. The control serum samples were collected from a group of 20 age paired healthy women. Informed consent was obtained from all the participants. Ovarian tissue samples (1 × 1 cm in size) were collected at the time of surgery. The samples were immediately snap‐frozen in liquid nitrogen and maintained at −80 °C until RNA extraction was performed.

**Table 1:** The clinical baseline characteristic of ovarian cancer patients

Variable	Value
Age, mean ± SD (years)	51.5 ± 15.4
Histological type, *n* (%)	36
Serous	23 (63.9)
Mucinous	4 (11.1)
Endometrioid	5 (13.9)
Clear cell	4 (11.1)
Differentiation, *n* (%)	36
G1	8 (22.2)
G2	15 (41.7)
G3	13 (36.6)
Stage, *n* (%)	36
I	5 (13.9)
II	14 (38.9)
III	15 (41.7)
IV	2 (5.6)
Lymphatic metastasis, *n* (%)	36
Negative	30 (83.3)
Positive	6 (16.7)

### Cell line and cell culture

SKOV3 ovarian cancer cell lines were obtained from the China Infrastructure of Cell Line Resources (Beijing, China) and cultured in Dulbecco's modified Eagle's medium containing 10% fetal bovine serum (Hyclone, Logan, UT, USA), 100 IU·mL^−1^ penicillin and 10 mg·mL^−1^ streptomycin. All cells were maintained at 37 °C under an atmosphere of 5% CO_2_.

### Serum exosome extraction

Serum exosomes were extracted using ExoQuick Exosome Precipitation Solution (System Biosciences, Mountain View, CA, USA) in accordance with the manufacturer's instructions. Briefly, serum samples were obtained by centrifugation at 3000 ***g*** for 15 min to remove cells and cellular fragments, and subsequent filtration of the supernatant was accomplished through a 0.45 μm pore polyvinylidene fluoride filter (Millipore, Billerica, MA, USA). ExoQuick was added to the supernatants and incubated for 12 h at −20 °C. Exosome pellets were collected after centrifugation at 1500 ***g*** for 30 min and then dissolved in 20 μL of PBS.

### RNA isolation and quantitative RT‐PCR

Total RNA was extracted from exosomes or from 50 mg tissue samples using Trizol Reagent (Invitrogen, Carlsbad, CA, USA) in accordance with the manufacturer's instructions. The expression of miRNAs was detected by quantitative PCR using a TaqMan miRNA probe. Briefly, single‐stranded cDNA was obtained using a TaqMan miRNA Reverse Transcription Kit (Applied Biosystems, Foster City, CA, USA) and then quantified using TaqMan Universal PCR Master Mix together with miRNA‐specific TaqMan MGB probes (Applied Biosystems) after 1 : 15 dilution. The U6 level was quantified for normalization. The experiment was repeated three times with detection in triplicate for each sample in each group.

### Dual luciferase assay

A segment of 566 bp brain‐derived neurotrophic factor (BDNF) 3′‐UTR was cloned into pmirGLO plasmid, downstream of the firefly luciferase coding region (Promega, Madison, WI, USA) to generate luciferase reporter vector. For luciferase reporter assays, SKOV3 cells were seeded in 48‐well plates. miRNAs mimic or inhibitor was transfected into SKOV3 cells with luciferase reporter vector using Lipofectamine 3000 (Invitrogen). Two days after transfection, cells were harvested and assayed with the Dual‐Luciferase Assay kit (Promega). The experiment was repeated three times with detection in triplicate for each sample in each group. The results were expressed as relative luciferase activity (firefly luciferase activity/renilla luciferase activity).

### Western blotting

Extracted protein samples were first quantified using a BCA Protein Assay Kit (Fisher Scientific Co., Pittsburgh, PA, USA) and then boiled in SDS/β‐mercaptoethanol sample buffer. Samples (20 μg) were loaded into each lane of 10% polyacrylamide gels and then the proteins were separated by electrophoresis. Subsequently, the proteins were transferred onto poly(vinylidene difluoride) membranes (Amersham Pharmacia Biotech, St Albans, Herts, UK) by electrophoretic transfer. After 1 h of incubation with 5% BSA, the membrane was incubated with rabbit anti‐BDNF monoclonal antibody (Abcam, Cambridge, MA, USA) or mouse anti‐β‐actin monoclonal antibody (Santa Cruz Biotechnology Inc., Santa Cruz, CA, USA) overnight at 4 °C. The specific protein–antibody complex was detected using horseradish peroxidase conjugated goat anti‐rabbit or rabbit anti‐mouse IgG. Detection by the chemiluminescence reaction was carried out using an ECL kit (Pierce, Appleton, WI, USA). The β‐actin signal was used as a loading control.

### Cell migration and invasion assay

Typical 24‐well Transwell plates (diameter 6.5 mm, pore size 8 μm; Costar Inc., Cambridge, MA, USA) were used for migration and invasion assays. Briefly, 3 × 10^4^ SKOV3 cells were seeded to the top chamber in 200 μL of serum‐free medium and 500 μL of medium with 5% serum was added to the bottom. After 12 h of incubation, filters were then submerged in 4% paraformaeldehyde for 15 min and cells on the upper surface were removed by cotton swabs. The cells on the lower surface were then stained by hematoxylin and eosin. Ten random fields were selected to determine the average number of migrated cells per view field.

For cell invasion assays, the procedure was similar to the cell migration assay, except Transwell membranes were pre‐coated with 24 μg·μL^−1^ Matrigel (BD Biosciences, Franklin Lakes, NJ, USA) and the cells were incubated for 24 h at 37 °C in a 5% CO_2_ atmosphere, with 10% serum medium in the bottom chamber. Cells adhering to the lower surface were counted the same way as the cell migration assay.

### Statistical analysis

All the results were analyzed using spss, version 16 (SPSS Inc., Chicago, IL, USA). Data were analyzed using Student's *t*‐test. *P* < 0.05 was considered statistically significant.

## Results

**Figure 1:**
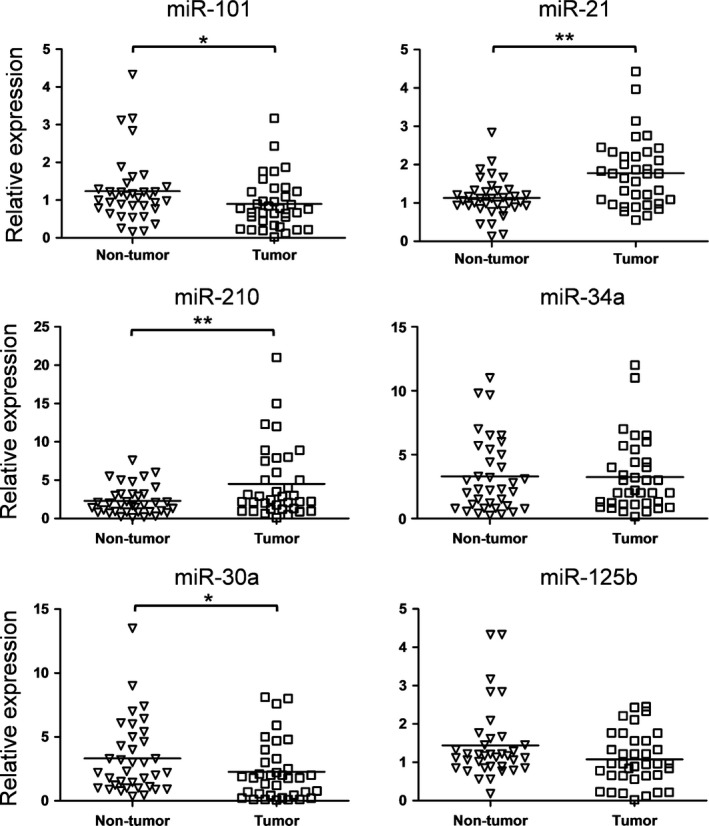
miRNA levels in ovarian cancer and adjacent nontumor control samples. Total RNA was extracted from ovarian cancer and adjacent nontumor control samples from 36 ovarian patients. The miRNA level was determined by quantitative RT‐PCR. The results were analyzed by Student's *t*‐test and *P* < 0.05 was considered statistically significant. *P < 0.05; **P < 0.01.

**Figure 2:**
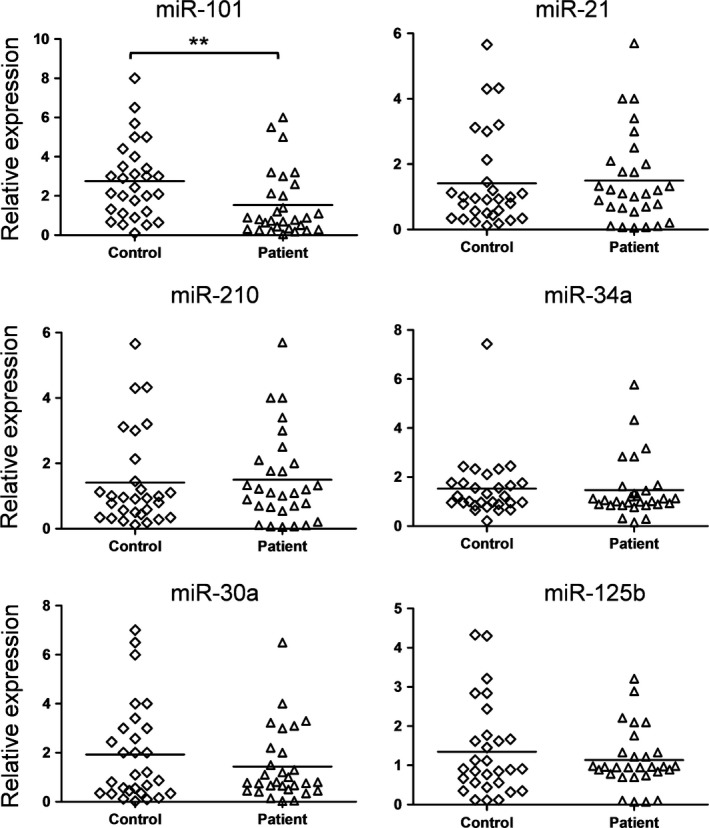
miRNA levels in exosomes from serum samples from ovarian cancer patients and healthy controls. Total RNA was extracted from the serum exosomes from 20 ovarian cancer patients and age paired controls. The miRNAs level was determined by quantitative RT‐PCR. The results were analyzed by Student's *t*‐test and *P* < 0.05 was considered statistically significant. **P < 0.01.

To investigate the role of miRNAs during the pathogenesis of ovarian cancer and to find a new biomarker for the diagnosis of ovarian cancer, we detected the expression of six candidate miRNAs in the tissue samples from 36 ovarian cancer patients. Meanwhile, the levels of these six candidate miRNAs in the serum exosomes from 20 age paired ovarian cancer patients and healthy controls were also examined. The six candidate miRNAs have been reported to be related to the carcinogenesis of ovarian cancer 12, 13, 14, 15. As shown in Fig. 1, the expression of miR‐101 and miR‐30a decreases and the expression of miR‐21 and miR‐210 increases in the tumor tissue samples compared to the nontumor controls. Only the expression of miR‐101 decreased significantly in the serum exosomes from ovarian cancer patients, suggesting a special role of miR‐101(Fig. 2).

**Figure 3:**
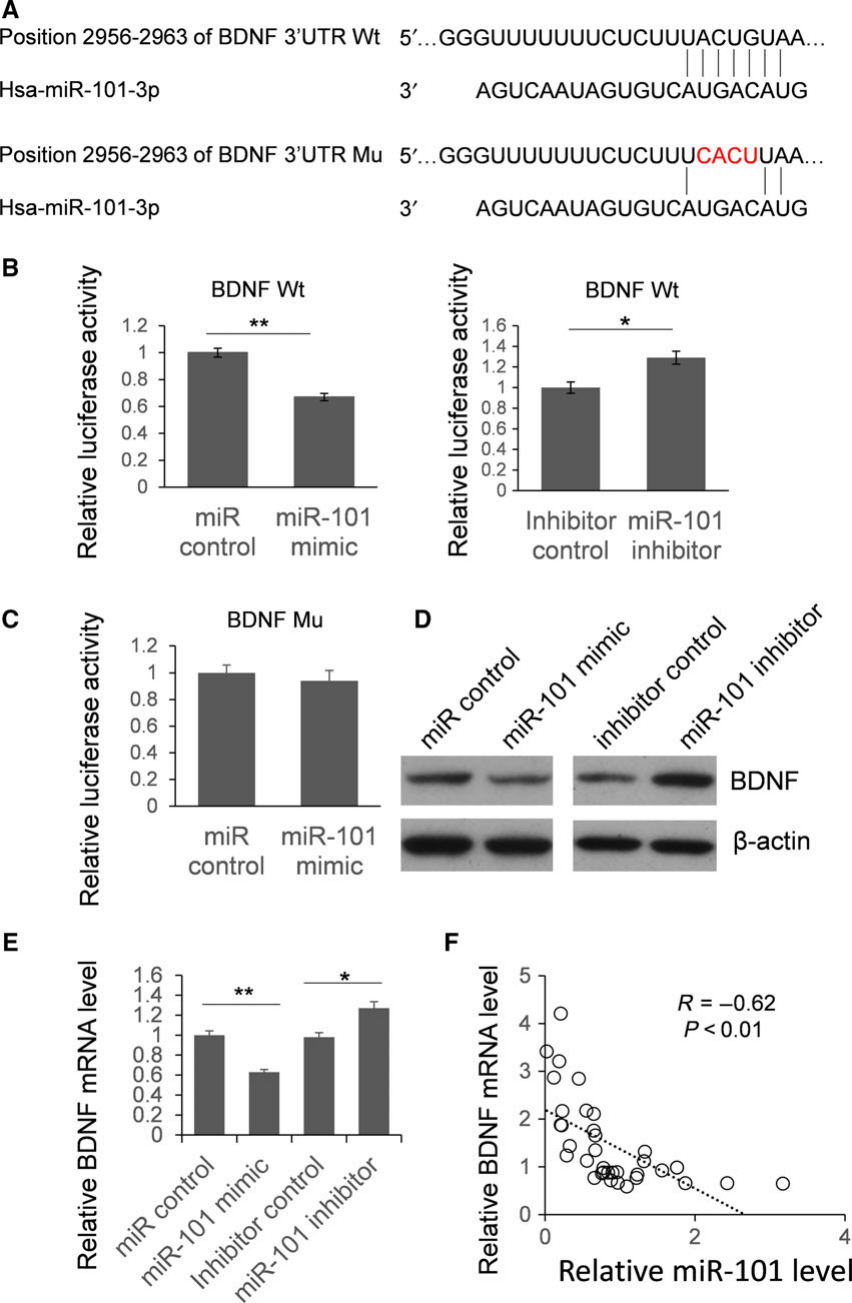
BDNF is a direct target of miR‐101. (A) Schematic diagram of the predicted interaction between miR‐101 and BDNF miRNA. (B,C) Dual luciferase assay to examine the direct interaction between and miR‐101 and BDNF. The results were analyzed by Student's *t*‐test and *P* < 0.05 was considered statistically significant. *P < 0.05; **P < 0.01. (D) Western blotting. SKOV3 cells were transfected by miR‐101 mimic or inhibitor. Forty‐eight hours after transfection, the cells were lysed and the protein level of BDNF was determined by immunoblotting. (E) SKOV3 cells were transfected with miR‐101 mimic or inhibitor for 48 h. Total RNA was extracted and the BDNF mRNA level was examined by quantitative RT‐PCR. The results were analyzed by Student's *t*‐test and *P* < 0.05 was considered statistically significant. *P < 0.05; **P < 0.01. (F) The level of BDNF mRNA in the tumor samples from 36 patients with OA was detected by quantitative RT‐PCR, and the correlation between BDNF and miR‐101 was analyzed using a chi‐squared test.

To explore the functional role of miR‐101 during the carcinogenesis of ovarian cancer, we predicted the target genes of miR‐101 using an online bioinformatics tool: targetscan (http://www.targetscan.org). We also constructed a mutant vector with four nucleotides mutation to confirm the target region of miR‐101(Fig. 3A). A dual luciferase assay was employed to identify the direct interaction between miR‐101 and the 3′‐UTR of BDNF. As shown in Fig. 3B, the relative luciferase activity was significantly reduced in the cells transfected with miR‐101 mimic compared to the control. Meanwhile, the luciferase activity was up‐regulated by miR‐101 inhibitor transfection. When four nucleotides changed, the luciferase activity was not repressed by miR‐101 mimic (Fig. 3C). These results indicate that miR‐101 represses luciferase expression by targeting the 3′‐UTR of BDNF.

To examine whether endogenous BDNF expression is modulated by miR‐101, SKOV3 cells were transfected with miR‐101 mimic or inhibitor. Forty‐eight hours after transfection, cells were lysed and the protein level of BDNF was examined by immunoblotting. As shown in Fig. 3D, the expression of BDNF decreased in the miR‐101 mimic transfected cells and increased in the miR‐101 inhibitor treated cells. To further understand miR‐101 induce BDNF mRNA degradation or translation retardation, the BDNF RNA level of SKOV3 cells transfected with miR‐101 mimic or inhibitor was examined by quantitative RT‐PCR. As shown in Fig. 3E, the BDNF mRNA level was reduced by miR‐101 mimic and up‐regulated by miR‐101 inhibitor, which indicated that miR‐101 repressed BDNF expression by inducing mRNA degradation. Meanwhile, the level of BDNF mRNA and miR‐101 in the tumor samples from 36 patients with OA underwent correlation analysis, and a significant negative correlation was found between miR‐101 and the BDNF RNA level (Fig. 3F).

**Figure 4:**
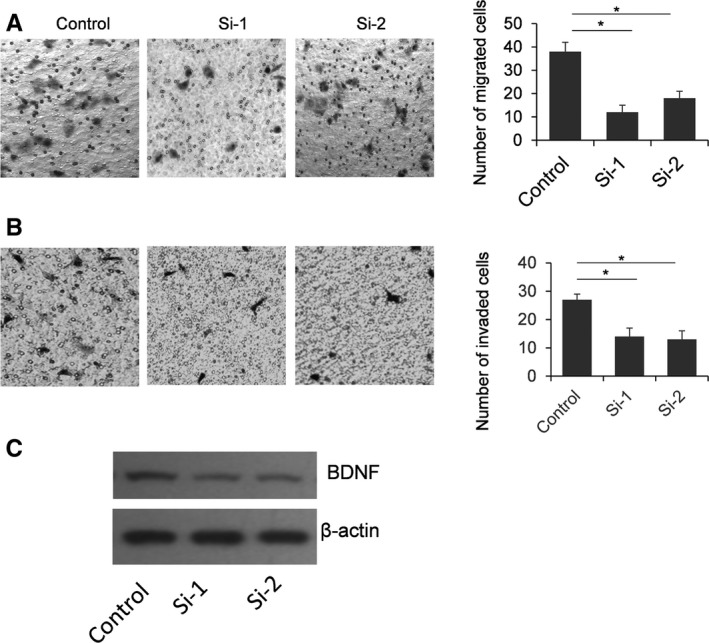
BDNF repression reduces cell migration and invasion ability. SKOV3 cells were transfected with Si‐1 or Si‐2 targeting BDNF for 48 h, with sequence scrambled short RNA oligo as a control. A typical Transwell assay was used to detect migration (A) and invasion (B) capacity of SKOV3 cells. The cells on the lower surface were stained with hematoxylin and eosin. Ten random fields were selected to determine the average number of cells per view field. The results were analyzed by Student's *t*‐test and *P* < 0.05 was considered statistically significant. *P < 0.05. The protein level of BDNF was examined by immunoblotting (C).

**Figure 5:**
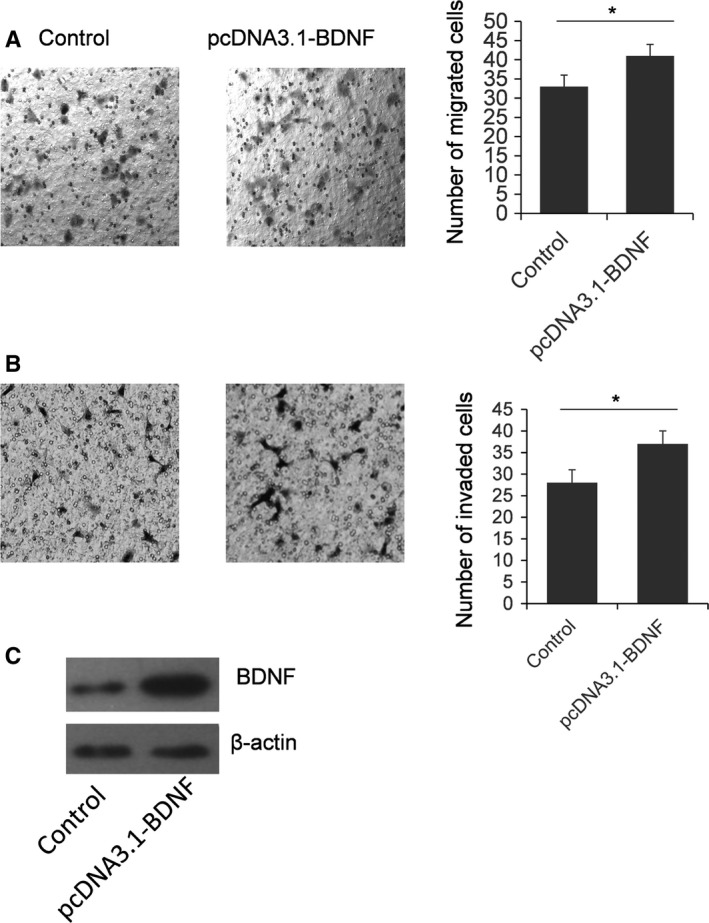
BDNF overexpression increases cell migration and invasion ability. SKOV3 cells were transfected with BDNF overexpression vector for 48 h, with empty vector as a control. A typical Transwell assay was used to detect migration (A) and invasion (B) capacity of SKOV3 cells. The cells on the lower surface were stained with hematoxylin and eosin. Ten random fields were selected to determine the average number of cells per view field. The results were analyzed by Student's *t*‐test and *P* < 0.05 was considered statistically significant. *P < 0.05 (A, B). The protein level of BDNF was examined by immunoblotting (C).

**Figure 6:**
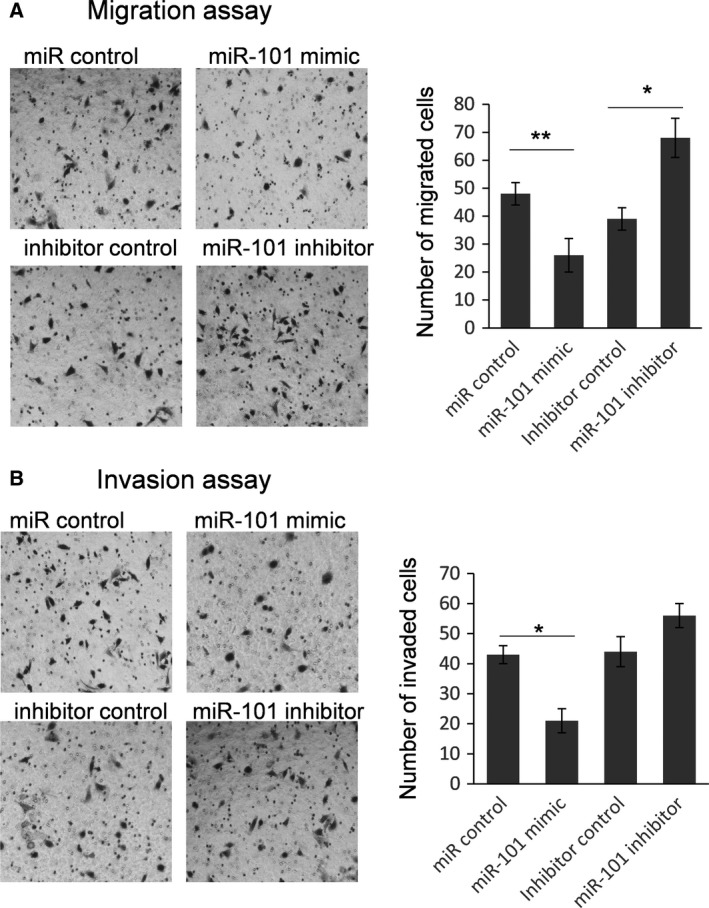
miR‐101 represses migration and invasion capacity of ovarian cancer cells. A typical Transwell assay was used to detect the function of miR‐101 with respect to migration (A) and invasion (B) capacity of SKOV3 cells. The cells on the lower surface were stained with hematoxylin and eosin. Ten random fields were selected to determine the average number of cells per view field. The results were analyzed by Student's *t*‐test and *P* < 0.05 was considered statistically significant. *P < 0.05; **P < 0.01.

Because the biological function of BDNF is mainly related to the migration and invasion of cancer cells, we examined the function of miR‐101 in SKOV3 cells on migration and invasion by a Transwell assay 15. A gain and loss function study of BDNF with respect to cell migration and invasion was conducted. As shown in Fig. 4, the number of migrated and invaded SKOV3 cells was significantly reduced when BDNF was knocked down. Meanwhile, the number of migrated and invaded SKOV3 cells was increased in the cells BDNF overexpressed, indicating that BDNF can promote the migration and invasion capacity in SKOV3 cells (Fig. 5). Finally, the number of migrated cells was reduced significantly by miR‐101 transfection. The miR‐101 inhibitor can significantly up‐regulate the number of migrated cells (Fig. 6A). As shown in Fig. 6B, miR‐101 can significantly repress SKOV3 cell invasion; however, only a slight increase of cell number is found in miR‐101 inhibitor treated cells.

## Discussion

Ovarian cancer is one of the deadliest gynecological tumors and both early‐stage detection and effective treatment methods are limited at present. In the present study, we first detected the expression of six candidate miRNAs in ovarian cancer tissues and adjacent nontumor ovarian samples from 36 patients and confirmed the disturbance of four miRNAs. Subsequently, the level of six candidate miRNAs was also examined in exosomes from patient serum samples. Only the level of miR‐101 was altered both in the ovarian tissue samples and serum exosomes. As predicted by bioinformatics tools and confirmed by dual‐luciferase assay and immunoblotting, we showed that miR‐101 can repress the expression of BDNF by targeting 3′‐UTR. Subsequently, we examined the function of miR‐101 on the migration and invasion capacity of ovarian cancer cells by a Transwell assay. The results obtained indicated that the reduction of miR‐101 is mostly related to significant enhanced ovarian cancer cell migration. Thus, the results of the present study indicate that the miR‐101 level in exosomes from serum has the potential for ovarian cancer diagnosis and that miR‐101 mimic is a potential therapeutic drug for ovarian cancer treatment.

Exosomes are a type of small extracellular vesicle proposed to be involved in a mechanism by which secreted cells pass signals to targeted cells. Tumor‐derived exosomes have a package with a special composition differing from the original cells and play direct or indirect roles in angiogenesis, immune escape and microenvironment modulation, and so on 16, 17, 18. In the present study, we report a miR‐101 reduction in the serum exosomes of ovarian patients. MiR‐101 reduction has the potential for clinical ovarian cancer diagnosis and classification, although the quantification of miR‐101 in different groups of patients needs to be processed first.

miRNAs comprise a group of versatile functional molecules that can modulate the signaling of several pathways by targeting multiple genes directly and indirectly. MiR‐101 has been confirmed to be a cancer repressor in a broad range of cancers, including lung cancer, breast cancer, liver cancer and bladder cancer 19, 20, 21, 22. In ovarian cancer, miR‐101 has been found directly repress EZH2, MARCH7, SOCS2 and ZEB1/2 to repress tumor cell proliferation, migration and invasion 14, 23, 24, 25. In the present study, we demonstrated that miR‐101 can repress ovarian cancer cell migration and invasion by blocking BDNF/TrkB signaling, which is an important supplement to previous research.

In conclusion, the present study has confirmed a correlation between miR‐101 reduction and ovarian cancer pathogenesis and this may provide a new biomarker for the clinical diagnosis and treatment of ovarian cancer.

## Author contributions

YX conducted the experiment, SZ conceived the project and fund the research. LX, JZ and LG participated the project.
